# Truncating *TINF2* p.Tyr312Ter variant and inherited breast cancer susceptibility

**DOI:** 10.1007/s10689-022-00295-z

**Published:** 2022-05-20

**Authors:** Susanna Koivuluoma, Sandra Vorimo, Tiina M. Mattila, Anna Tervasmäki, Timo Kumpula, Outi Kuismin, Robert Winqvist, Jukka Moilanen, Tuomo Mantere, Katri Pylkäs

**Affiliations:** 1 Laboratory of Cancer Genetics and Tumor Biology, Cancer and Translational Medicine Research Unit and Biocenter Oulu, NordLab Oulu, University of Oulu, 90220 Aapistie 5A, Oulu, Finland; 2 Department of Clinical Genetics, Medical Research Center Oulu and PEDEGO Research Unit, Oulu University Hospital, University of Oulu, Oulu, Finland

**Keywords:** Breast cancer, Hereditary predisposition, TINF2, Dyskeratosis congenita

## Abstract

TINF2 is a critical subunit of the shelterin complex, which protects and maintains the length of telomeres. Pathogenic missense and truncating *TINF2* mutations are causative for dyskeratosis congenita (DC), a rare, dominantly inherited bone marrow failure syndrome characterized by mucocutaneous abnormalities and cancer predisposition. Recent reports indicate that specific *TINF2* truncating mutations act as high penetrance cancer predisposition alleles outside DC context, including breast cancer in their tumor spectrum. Here, we have evaluated the role of germline mutations in *TINF2* and other shelterin genes in inherited breast cancer susceptibility using exome sequencing data from 98 Northern Finnish breast cancer cases with indication of inherited disease predisposition as a discovery cohort. A single protein truncating variant, *TINF2* p.Tyr312Ter, was identified in one of the cases (1/98), and four more carriers were observed in the subsequently genotyped unselected breast cancer cohort (4/1904). None of the carriers were reported to have DC. *TINF2* p.Tyr312Ter resulted in stable short form of mRNA transcript, and normal telomere length has been indicated by a recent report. Although recurrent in cases (total of 5/2095), *TINF2* p.Tyr312Ter is also present in Finnish population controls (8/12,517), and the observed 4-fold higher frequency in cases falls at most into the range of moderate breast cancer risk alleles (OR 3.74, 95% CI 1.22–11.45, p = 0.029). Current results indicate that not all *TINF2* truncating variants are high cancer risk alleles and add further evidence that different *TINF2* mutations can have very diverse effects on the disease phenotype.

**Supplementary information:**

The online version contains supplementary material available at 10.1007/s10689-022-00295-z.

## Introduction

*TINF2* (TERF1 Interacting Nuclear Factor 2) is a critical subunit of the shelterin complex, which protects telomere ends and maintains the telomere length by cooperating with telomerase [1]. The shelterin complex allows cells to differentiate between telomeres and damaged DNA by blocking the activation of double-strand break repair pathways and prohibiting hyper-resection at telomeres [2]. The shelterin complex consists of six proteins, TINF2, TERF1, TERF2, TERF2IP, ACD, and POT1, of which TINF2 and ACD mediate the complex assembly [1]. Pathogenic missense [3] and truncating mutations [4] of *TINF2* are causative for dyskeratosis congenita (DC), a rare, dominantly inherited bone marrow failure syndrome characterized by mucocutaneous abnormalities and cancer predisposition, most commonly head and neck cancers, anorectal squamous cell carcinoma, and stomach and lung cancer [5]. At cellular level, these *TINF2* mutations result in short telomere length, which is a molecular hallmark for DC caused by defects in telomere maintenance [3].

Recently, a truncating germline *TINF2* mutation, p.Trp198fs, was reported in a large family with several papillary thyroid carcinoma and melanoma cases, and it showed complete disease co-segregation. The telomere length was significantly longer in the identified mutation carriers, which is contrary to that previously reported in DC patients [6]. Following report revealed another two *TINF2* truncating mutations, p.Glu202fs/p.Leu170fs and p.Ser186fs, in altogether four cancer-prone families, where six *TINF2* mutation carriers developed numerous malignancies, including three papillary thyroid carcinomas, two melanomas and three breast carcinomas. In all four families, confirmed mutation carriers had at least one first-degree relative with breast cancer. At cellular level, both *TINF2* mutations also resulted in excessive telomere elongation [7]. Based on these reports, *TINF2* acts as a haploinsufficient tumor suppressor also outside the DC context, and specific truncation mutations resulting in long germline telomeres cause inherited cancer predisposition with high penetrance and severity [6, 7].

Here, we have evaluated the role of *TINF2* and other shelterin complex gene mutations in inherited breast cancer susceptibility. As a result, we report a recurrent *TINF2* truncation mutation, p.Tyr312Ter, which based on case-control comparisons is at highest a moderate-risk allele for breast cancer. This adds yet another feature for *TINF2* germline mutations that seem to result in remarkably distinct outcomes.

## Materials and methods

### Identification of rare truncating variants in shelterin complex genes

Variant calls from exome sequencing data of 98 Northern Finnish breast cancer cases with indication of hereditary disease susceptibility [8] were assessed for rare and potentially deleterious variants in the six shelterin complex genes: *TINF2*, *TERF1*, *TERF2*, *TERF2IP*, *ACD*, and *POT1*. The filtering criteria used was: (1) inclusion of variants with predicted protein truncations (nonsense, frameshift and splice site variants) and (2) inclusion of variants with minor allele frequency, MAF < 0.01 in dbSNP, Ensembl, GnomAD and SISu databases. This discovery cohort included (1) index cases from families with 3 ≤ breast and/or ovarian cancer cases in 1st or 2nd degree relatives (n = 83), (2) index cases from families with two cases of breast, or breast and ovarian cancer in 1st or 2nd degree relatives, of which at least one with early disease onset (< 35 years), bilateral disease or multiple primary tumors (n = 7), and (3) breast cancer cases diagnosed at or below the age of 40 (n = 8).

### Variant genotyping in additional cohorts

One variant, *TINF2* p.Tyr312Ter, passing the filtering was genotyped further in breast cancer cohorts collected from same geographical area, defined as hereditary (n = 93) and unselected breast cancer cohort (n = 1904). The hereditary cohort (n = 93) consisted of *BRCA1/2* and *PALB2* mutation negative breast cancer index cases from (1) 44 families with 3 ≤ breast cancer cases in 1st or 2nd degree relatives, (2) 17 families with two cases of breast cancer in 1st or 2nd degree relatives, of which at least one had early disease onset (< 35 years), bilateral disease or multiple primary tumors including breast cancer, and (3) 32 families with two cases of breast cancer in 1st or 2nd degree relatives. The unselected cohort consisted of 1904 consecutive breast cancer cases diagnosed at the Oulu University Hospital during the years 2000–2019, unselected for their family history of cancer and age at disease onset. Tumor characteristics for the cases were obtained from pathology reports. This study included informed consent from all participating individuals. Genotyping was done using high-resolution melt analysis (CFX96, Bio-Rad) and Sanger sequencing (ABI3130xL, Applied Biosystem). Data from Finnish individuals (GnomAD, https://gnomad.broadinstitute.org/ and SISu, http://www.sisuproject.fi/) was used as control for comparisons.

### Statistical analyses

Carrier frequencies between cases and controls were compared using Fisher’s exact test (IBM SPSS Statistics 26.0 for Windows). All p-values were two-sided.

### Transcript analysis

RNA was extracted from patient-derived lymphoblastoid cell-lines using RNeasy Mini Kit (Qiagen) and reverse transcribed into cDNA with iScript cDNA synthesis Kit (Bio-Rad). PCR amplified (Table S1) fragments targeting both short and long isoforms were separated on agarose gel, extracted using UltraClean GelSpin DNA Extraction Kit (MO BIO) and Sanger sequenced (ABI3500xL).

## Results

**Fig. 1:**
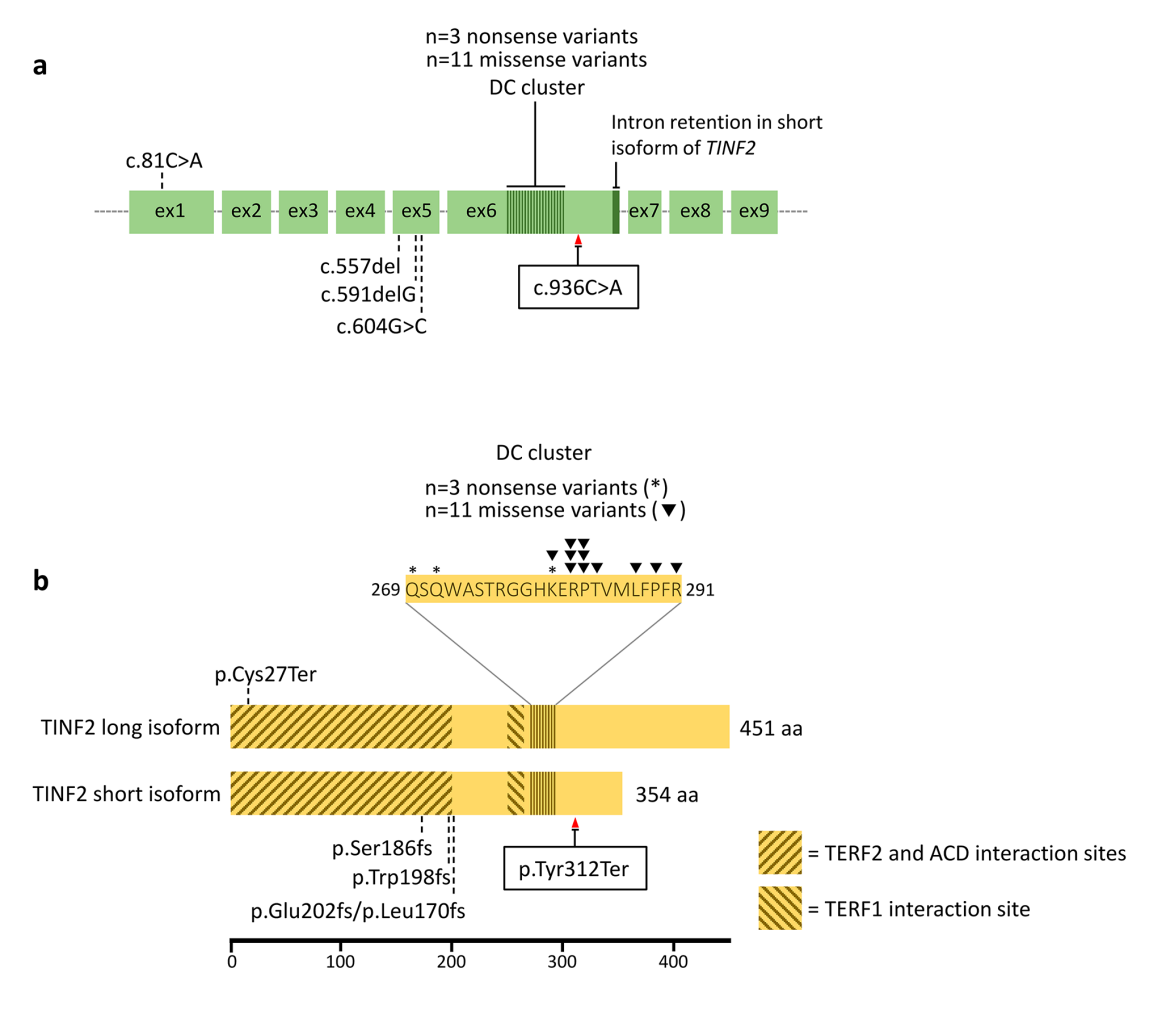
Schematic presentation of *TINF2* (a) gene and (b) protein, and localization of currently identified and previously discovered pathogenic variants for both DC and high cancer risk. TINF2 short isoform is a result of a small intron retention between exon 6 and 7 and the consequent stop codon [9]. Based on the cDNA analysis, c.936 C > A variant is stable only in this mRNA isoform. In exon 5, truncating variant c.591delG (p.Trp198fs) is associated with papillary thyroid carcinoma and melanoma [6], and a splice donor variant c.604G > C (with predicted truncations p.Glu202fs and p.Leu170fs) and c.557del (causing frameshift and a stop codon, p.Ser186fs) are proposed as high-risk alleles for multiple cancer types [7]. A majority of missense and truncating variants associated with DC localize to exon 6, specifically to a highly conserved area called DC cluster [3]. Variants associated with high cancer risk are shown under schematic gene and protein illustrations, and DC variants above (ClinVar database, https://www.ncbi.nlm.nih.gov/clinvar/). Variant c.936 C > A (p.Tyr312Ter) is pointed by a red arrow. TERF2, ACD and TERF1 interaction sites are marked with diagonal stripes

Analysis of 98 breast cancer cases for shelterin complex gene mutations (Table S2) revealed only one protein truncating variant, *TINF2* p.Tyr312Ter (c.936 C > A, rs201677741). The carrier was diagnosed with breast cancer at the age of 27 years and had both breast and ovarian cancer in 3rd degree relatives (Table S3). Although there were no additional DNA samples from the family to study the disease segregation, the position and mutation type of the variant suggested that it could be a pathogenic mutation analogous to other previously reported variants in the gene (Fig. 1). To support this, the variant was globally rare with MAF of 0.0003 in European populations (both non-Finnish and Finnish, GnomAD), and had also equivalent MAF (0.00038) in Northern Finland, Northern Ostrobothnia (SISu).

*TINF2* p.Tyr312Ter was genotyped in further breast cancer case cohorts, and the variant prevalence is summarized in Table 1. No *TINF2* p.Tyr312Ter carriers were identified in the additional cohort of *BRCA1*/*2* and *PALB2* mutation negative index cases with indication of inherited background for the disease. However, four more carries were identified from the unselected breast cancer cohort (4/1904, 0.21%, OR = 3.29, 95% CI = 0.99–10.49, p = 0.063). The mean age at disease onset for the unselected carriers was 61.3 years, which is similar to the mean for this cohort (58 years, range 28–93 years). When all breast cancer cases (5/2095, 0.24%) were compared to carriers reported in Finnish population (8/12,517, 0.06%), the frequency was 4 times higher with OR 3.74 (95% CI 1.22–11.45, p = 0.029).

**Table 1:** Frequency of *TINF2* p.Tyr312Ter in breast cancer cases and controls

Cohort	N	WT	%	Mut	%	OR	95% CI	p
Hereditary Br^a^	191	190	99.48	1	0.52	8.23	1.02–66.12	0.127
Unselected Br	1904	1900	99.79	4	0.21	3.29	0.99–10.49	0.063
All Br	2095	2090	99.76	5	0.24	3.74	1.22–11.45	0.029
Controls^b^	12,517	12,509	99.94	8	0.06			

Br = Breast cancer, CI = Confidence interval, Mut = Mutation carrier, OR = Odds ratio, WT = Wild-type

^a^ 98 exome-sequenced and 93 additionally genotyped index cases

^b^ GnomAD European (Finnish) population

All four *TINF2* p.Tyr312Ter carriers from the unselected cohort had family history of cancer, but only one of the families showed cancer types (one melanoma case) previously linked to *TINF2*-related cancer susceptibility [7]. Unfortunately, there were no samples available to study the mutation’s co-segregation with the cancer phenotype in the respective families (Table S3). The breast tumor characteristics of the *TINF2* p.Tyr312Ter carriers are summarized in Table S4. Based on the reported cancer history and other diseases in the family, there were no indications of DC for any of the carriers or their family members.

The effect of the *TINF2* p.Tyr312Ter variant at transcriptional level was studied with mRNA specific sequencing. The presence of the variant was confirmed only in the short form of *TINF2* transcript, where it resides in the last coding exon (6/6) of the transcript and the created premature stop codon abolishes the last 42 amino acids of the short protein isoform. It was absent from the long transcript likely due to nonsense-mediated decay targeting the isoform with premature stop codon in exon 6/9 (Fig. 1, Fig. S1).

## Discussion

Specific truncating mutations of *TINF2* that encodes an integral member of the shelterin complex, critical for telomere protection, have previously been reported to cause a high risk for cancer [6, 7]. To evaluate the role of germline mutations in *TINF2* and other shelterin encoding genes in inherited breast cancer susceptibility, we analyzed exome sequencing data from 98 Northern Finnish breast cancer cases with indication of hereditary disease susceptibility. There were no truncating germline mutations in the *TERF1*, *TERF2*, *TERF2IP*, *ACD*, and *POT1* genes, whereas a single truncating variant in *TINF2*, p.Tyr312Ter, was observed.

Although *TINF2* p.Tyr312Ter variant was recurrent in breast cancer cases, it is also reported in public databases in apparently healthy individuals. Based on the case-control comparisons, the frequency of *TINF2* p.Tyr312Ter in breast cancer cases was 4-fold higher compared to controls, which falls at most into the range typical for moderate breast cancer risk alleles [10]. Even though *TINF2* p.Tyr312Ter variant locates near the DC cluster of the protein, none of the currently identified cases or their family members were reported to have any of the symptoms of this disorder. This might be due to variant localizing closer to the C-terminal end of the protein than the DC causing truncation mutations in the same exon and therefore the functionality of the produced truncated protein from the short transcript is retained. In support of this, there is a recent case report of *TINF2* p.Tyr312Ter variant carrier with normal telomere length. She suffered from herpes simplex virus 2 encephalitis but was otherwise healthy [11]. Thereby *TINF2* p.Tyr312Ter does not seem to result in either elongated or shortened telomeres, the cellular level features associated with *TINF2* mutations related to inherited high-risk cancer predisposition and DC, respectively.

In conclusion, the results indicate that rare variants in shelterin complex genes do not play a major role in inherited breast cancer susceptibility, at least in the current study population. The only observed truncating mutation was *TINF2* p.Tyr312Ter, and although it was more prevalent in cases compared to controls, it is unlikely a high cancer risk allele analogous to those *TINF2* truncation mutations previously described in the literature [6, 7]. Our results provide additional evidence that *TINF2* mutations in different gene positions can have remarkably diverse effects on individuals’ phenotype, ranging from severe congenital disorder to potentially moderately increased risk for breast cancer.

## Electronic supplementary material

Below is the link to the electronic supplementary material.

Supplementary Material 1

## Data Availability

The data to support the findings of this study is available on request from the corresponding author.
