# Supplementary material for: Truncating TINF2 p.Tyr312Ter variant and inherited breast cancer susceptibility
Source: Fam Cancer. 2022 May 20;22(1):13–7. doi: 10.1007/s10689-022-00295-z (PMC9829577; doi:10.1007/s10689-022-00295-z)
Supplement: Supplementary file 1 — Supplementary Material 1 [file 10689_2022_295_MOESM1_ESM.pdf]

Truncating *TINF2* p.Tyr312Ter variant and inherited breast cancer susceptibility

Supplementary data

Susanna Koivuluoma, MSc[1], Sandra Vorimo, BSc[1], Tiina M. Mattila, PhD[1], Anna Tervasmäki, PhD[1], Timo Kumpula, MSc[1], Outi Kuismin, MD, PhD[2], Robert Winqvist, PhD[1], Jukka Moilanen, MD, PhD[2], Tuomo Mantere, PhD[1], Katri Pylkäs, PhD[1, *]

[1]Laboratory of Cancer Genetics and Tumor Biology, Cancer and Translational Medicine Research Unit and Biocenter Oulu, NordLab Oulu, University of Oulu, Oulu, Finland

[2]Department of Clinical Genetics, Oulu University Hospital, Medical Research Center Oulu and PEDEGO Research Unit, University of Oulu, Oulu, Finland

Table S1 cDNA-specific PCR primers used for the amplification of short and long TINF2 isoforms

	Short isoform	Long isoform
**Forward primer**	GACAATATGGTGTGGACATGG	GACAATATGGTGTGGACATGG
**Reverse primer**	GCAAGCAAAGAGGATAGGATG	GCCAGAAGGGGGTAGGTATT

Table S2 Results from exome data analysis filtered for rare (MAF< 0.01) missense and protein truncating variants (PTV) in shelterin complex genes

	Genomic location (Hg19)[a]	PTV	Missense
TINF2	chr14:24,708,846-24,711,880	p.Tyr312Ter[b]	None
TERF1	chr8:73,921,099-73,960,358	None	p.Ala21Gly[c]
TERF2	chr16:69,389,470-69,419,910	None	None
TERF2IP	chr16:75,681,671-75,691,330	None	None
ACD	chr16:67,691,415-67,694,163	None	None
POT1	chr7:124,462,440-124,569,879	None	None

[a] Genomic coordinates from RefSeq

[b] *TINF2* p.Tyr312Ter (c.936C>A, rs201677741)

[c] *TERF1* p.Ala21Gly (c.62C>G, rs370999354)

Table S3 Family history of cancer in *TINF2* c.936C>A (p.Tyr312Ter) positive index cases

Index ID - Cancers/tumors (age at diagnosis)	Breast/ovarian cancer(s) in 1st and/or 2nd degree relatives (age at diagnosis)	Breast/ovarian cancer(s) in 3rd degree relatives (age at diagnosis)	Other cancers in 1st and/or 2nd degree relatives (age at diagnosis)	Other cancers in 3rd degree relatives (age at diagnosis)
**Her1 -Br (27)**	-	Br (u), Br + Ov (40)	-	Sto (u)
**Uns1 -Br (51)**	-	-	Lu (u), Csu (u), Csu (u)	-
**Uns2 -Br (64)**	Br (u), Ov (u)	-	Lu (56), Pro (65)	-
**Uns3 -Br (66)**	Br (u)	-	Pro (u), Es (60), Csu (u), Pro (u)	-
**Uns4 -Br (64)**	-	-	Mel[a] (u)	-

Br=Breast cancer, Csu=Cancer site unknown, Es=Esophagus, Her=hereditary cohort, Lu=Lung cancer, Mel=Melanoma, Ov=Ovarian cancer, Pro=Prostatic cancer, Sto=Stomach cancer, u=Unknown, Uns=unselected cohort.

[a] Sister of the index

Table S4 Tumor characteristics of *TINF2* c.936C>A p.Tyr312Ter carriers

Index ID	Morphology	Receptor status (ER, PR, HER2)	TNM
**Her1**	Ductal	ER-, PR-, HER2+	T2N1M1
**Uns1**	Ductal	ER+, PR+, HER2-	T2N1M0
**Uns2**	Ductal	ER+, PR+, HER2-	T1N0M0
**Uns3**	Lobular	ER+, PR+, HER2-	T1N0M0
**Uns4**	Ductal	ER+, PR+, HER2-	T1N0M0

Her=Hereditary cohort, Uns=Unselected cohort, T=Tumor size, N=Nodal status, M=Primary metastasis, ER=Estrogen receptor, PR=Progesterone receptor.

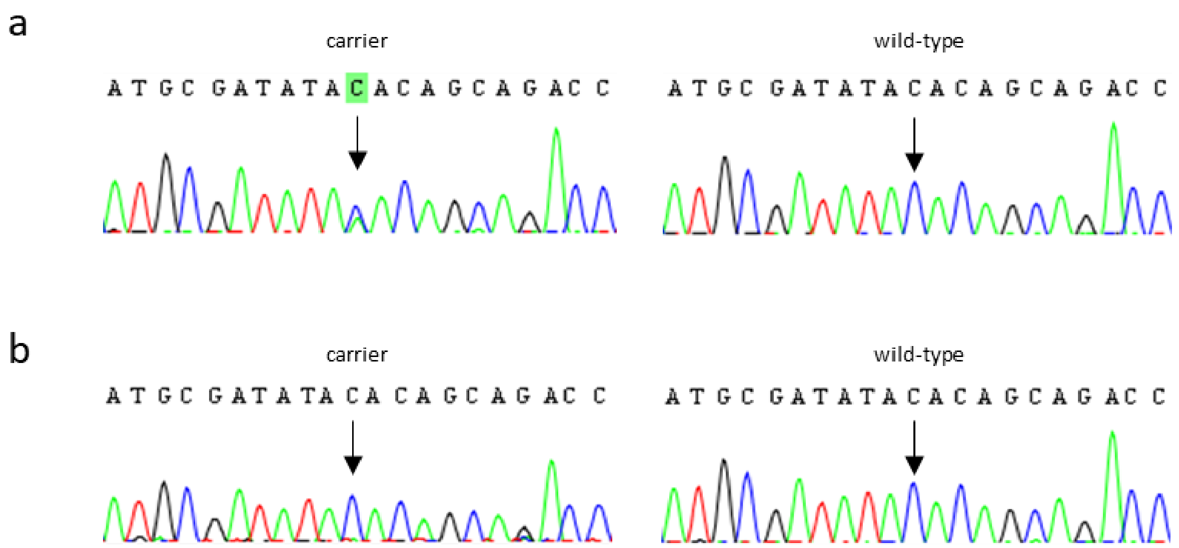

Fig. S1 Sequence chromatograms of *TINF2* c.936C>A (p.Tyr312Ter) variant carrier and wild-type in (a) the short isoform of *TINF2* cDNA and (b) the long isoform cDNA. Mutation site c.936C>A is pointed by black arrows.
